# Parent and patient knowledge and attitudes about cancer predisposition syndrome genetic testing in pediatric oncology: Understanding sociodemographic and parent–child differences

**DOI:** 10.1002/cnr2.2119

**Published:** 2024-09-05

**Authors:** Chelsea S. Rapoport, Diane Masser‐Frye, Sapna Mehta, Alyssa K. Choi, Sydney Olfus, Megan Korhummel, Veronica Hoyo, David Dimmock, Vanessa L. Malcarne, Dennis J. Kuo

**Affiliations:** ^1^ San Diego State University/University of California San Diego Joint Doctoral Program in Clinical Psychology La Jolla California USA; ^2^ Division of Genetics/Dysmorphology Rady Children's Hospital San Diego San Diego California USA; ^3^ Department of Global Health University of California San Diego La Jolla California USA; ^4^ Department of Biology San Diego State University San Diego California USA; ^5^ Department of Psychology San Diego State University San Diego California USA; ^6^ Altman Clinical and Translational Research Institute University of California San Diego La Jolla California USA; ^7^ Rady Children's Institute for Genomic Medicine San Diego California USA; ^8^ Department of Psychiatry University of California San Diego La Jolla California USA; ^9^ Department of Pediatrics University of California San Diego La Jolla California USA; ^10^ Division of Pediatric Hematology‐Oncology Rady Children's Hospital San Diego San Diego California USA

**Keywords:** genetic counseling, genetic testing, hereditary neoplastic syndromes, pediatric cancer, pediatric oncology, psychosocial studies, sociodemographic differences

## Abstract

**Background:**

Cancer predisposition syndromes (CPS) impact about 10% of patients with pediatric cancer. Genetic testing (CPS‐GT) has multiple benefits, but few studies have described parent and child knowledge and attitudes regarding CPS‐GT decision‐making. This study examined parent and patient CPS‐GT decision‐making knowledge and attitudes.

**Procedure:**

English‐ or Spanish‐speaking parents of children with pediatric cancer and patients with pediatric cancer ages 15–18 within 12 months of diagnosis or relapse were eligible to participate. Seventy‐five parents and 19 parent‐patient dyads (*N* = 94 parents, 77.7% female, 43.6% Latino/a/Hispanic; 19 patients, 31.6% female) completed surveys measuring CPS‐GT‐related beliefs. Independent samples *t*‐tests compared parent responses across sociodemographic characteristics and parent‐patient responses within dyads.

**Results:**

Spanish‐speaking parents were significantly more likely than English‐speaking parents to believe that CPS‐GT not being helpful (*p* < .001) and possibly causing personal distress (*p* = .002) were important considerations for deciding whether to obtain CPS‐GT. Parents with less than four‐year university education, income less than $75,000, or Medicaid (vs. private insurance) were significantly more likely to endorse that CPS‐GT not being helpful was an important consideration for deciding whether to obtain CPS‐GT (*p* < .001). Parents felt more strongly than patients that they understood what CPS‐GT was (*p* = .01) and that parents should decide whether patients under 18 should receive CPS‐GT (*p* = .002).

**Conclusions:**

Spanish‐speaking parents and parents with lower socioeconomic statuses were more strongly influenced by the potential disadvantages of CPS‐GT in CPS‐GT decision‐making. Parents felt more strongly than patients that parents should make CPS‐GT decisions. Future studies should investigate mechanisms behind these differences and how to best support CPS‐GT knowledge and decision‐making.

## 1 INTRODUCTION

Previous studies have shown that cancer predisposition syndromes (CPS) are a contributing cause in approximately 10% of pediatric cancer cases.^1^, ^2^ CPS result from the germline mutations that put individuals at increased risk for developing cancer at an earlier age compared to the general population. There are multiple benefits of genetic testing for CPS (CPS‐GT), including valuable information for clinical decision‐making, increased accessibility of genetically informed treatment interventions, better patient health outcomes, and opportunities for cancer surveillance programs for affected family members.^1^, ^3^, ^4^, ^5^ Despite these benefits, recommendations for CPS‐GT are not consistently applied or understood, suggesting a need for more standardized CPS‐GT guidelines across medical institutions.^6^

CPS‐GT decision‐making (i.e., deciding to partake in CPS‐GT) and results‐sharing for patients with pediatric cancer present challenging ethical issues given that parental consent is required for patients under the age of 18 in the US, but patient assent is not.^7^ Aside from legal restrictions, there is ongoing debate about the extent to which patients under the age of 18 should have autonomy in CPS‐GT decision‐making.^7^, ^8^

Parents generally agree that CPS‐GT is useful but express varying attitudes regarding making decisions about pediatric CPS‐GT.^5^, ^6^, ^8^ The limited literature suggests common parental motivations for CPS‐GT are to become more knowledgeable about treatment implications, understand the reasons behind the diagnosis, and delineate familial risk.^1^, ^4^, ^8^, ^9^ In contrast, parental perceptions of CPS‐GT disadvantages include concerns about stigma, distress, insurance discrimination, and misinterpretation or mistrust of results.^1^, ^4^, ^9^ Parents of children at risk of CPS‐GT due to family history believe parents should make the final decision for CPS‐GT, but report that they would want to have open conversations with their children.^8^, ^10^ The few studies of pediatric patient opinions regarding CPS‐GT decision‐making suggest that patients would like to be involved in decision‐making while still incorporating parental approval.^9^, ^11^ Some patients have also reported that CPS‐GT results were shared by their parents in unplanned conversations, which was associated with greater patient distress.^10^ Given that the literature is sparse, research is needed to elucidate patient and parent knowledge and attitudes on CPS‐GT. In particular, more research is needed on patient and parent knowledge and attitudes regarding CPS‐GT, as these can have major implications on future medical decision‐making for patients and family members.

Few studies have examined sociodemographic characteristics in relation to CPS‐GT‐related perceptions. Factors such as limited insurance coverage and lower income may serve as barriers to CPS‐GT and therefore may have important implications for decision‐making.^1^, ^12^ Further, literature suggests that sociodemographic variables, such as Black (vs. White) race are associated with declining pediatric oncology genome sequencing opportunities for families of patients with pediatric cancer.^12^ Conversely, studies in other medical populations (e.g., prenatal testing, BRCA testing) show that Latino/Hispanic and African American/Black patients have favorable views on GT and may even have greater interest in GT than non‐Latino/Hispanic and non‐African American/Black patients, despite facing greater barriers and lower factual knowledge.^13^, ^14^, ^15^, ^16^ A systematic review found that research in this area is limited and inconsistent in methodology, with few comparisons between White and non‐White participants.^17^ Literature is otherwise minimal on potential sociodemographic influences, such as insurance, education, or income, on CPS‐GT decision‐making.

The present pilot study surveyed parents of children/adolescents with confirmed cancer diagnoses, as well as parent‐patient dyads for patients ages 15 to 18, and had three primary aims: (1) to describe parent knowledge and attitudes about CPS‐GT; (2) to identify sociodemographic differences in parent CPS‐GT knowledge and attitudes; and (3) to compare parent and patient CPS‐GT knowledge and attitudes for parent‐patient dyads. This study will address an important gap in the literature regarding sociodemographic considerations in CPS‐GT beyond race and ethnicity, as well as patient versus parent knowledge and attitudes pertaining to CPS‐GT decision‐making, disclosure, and positive and negative impacts, which have yet to be explored comprehensively in a single study.

## 2 METHODS

This study was approved by UCSD's Human Research Protections Program (UCSD IRB #181972).

### 2.1 Participants

Patients with cancer in the pediatric oncology clinic at Rady Children's Hospital San Diego (RCHSD) who had been diagnosed with a new or relapsed malignancy within 12 months of study enrollment and their parents were recruited for the study. Parents were eligible to complete the surveys if their children were under the age of 18, and patients ages 15 to 18 were eligible to complete separate CPS‐GT surveys from their parents. All participants had to be English or Spanish‐speaking.

### 2.2 Procedure

Eligible parents and parent‐patient dyads (for patients ages 15–18) were approached by an oncologist to discuss the study during their medical appointments. Participants were recruited to a study that assessed knowledge and attitudes regarding CPS‐GT and provided access to genetic counseling, which included an offer of CPS‐GT if aligned with clinical standards of care. Options included single gene testing per the regular commercial clinical route for a CPS if the participant met standard clinical criteria for testing, or broader panel testing using the Invitae™ Multi‐Cancer Panel (https://www.invitae.com/en/physician/tests/01101/). This panel tests for genetic variants in over 82 genes that can be associated with both childhood and adult‐onset cancer predisposition. Written informed consent was obtained from parents and written assent was obtained from adolescents ages 15–18. If adolescents did not assent to participation, then they were not enrolled in the study. Pre‐March 2020, patients and parents completed their baseline, pre‐counseling survey on a tablet through REDCap at a medical appointment in the clinic exam room. After the start of the COVID‐19 pandemic due to infection control considerations, participants were sent the survey via email post‐consent. Parents (one per patient) and eligible patients completed surveys independently. This report describes baseline data of self‐reported knowledge and attitudes in parents and patients, prior to genetic counseling.

### 2.3 Measures

Prior to self‐report survey development, the study team reviewed the literature and determined that an assessment focusing on CPS‐GT in patients with pediatric cancer and their parents had not yet been developed. The study team, led by an expert in psychometric development (VH), then created a new measure modeled after measures in the field, incorporating items adapted from validated, reliable surveys on GT knowledge and attitudes and using previous literature to inform the number of items and response scale.^15^, ^16^, ^18^, ^19^, ^20^, ^21^, ^22^ The literature review aimed to identify major themes in the literature, order and number of items, and response scale. Specifically, this study drew several questions regarding knowledge and attitudes from the Pediatric BRCA1/2 Testing Attitudes Scale (P‐TAS), a reliable, valid measure (Cronbach's coefficient = .90) for women who had children and were participating in BRCA testing.^20^ Additionally, several questions on reasons for engaging in CPS‐GT and potential risks were drawn from a survey developed for medical practitioners regarding referrals to CPS‐GT and hereditary cancer clinics.^21^ These questions were developed to assess medical practitioner understanding of CPS risk factors and reasons for not referring patients for CPS‐GT. Once appropriate themes and items were identified, compared, and contrasted, the survey development team drafted survey questionnaires tailored to the research question. The surveys have not previously been validated in teenagers. Therefore, reviewers with clinical and research expertise with the target populations were consulted to determine face and content validity of the surveys. These reviewers provided feedback and the survey was revised accordingly. Final questionnaires were confirmed as readable through a consensus calculator (https://readabilityformulas.com/). Instruments were translated to Spanish by Rady Children's Hospital San Diego's translation service and reviewed for accuracy by a survey developer who is a native Spanish speaker and certified for translation in healthcare settings.

Questions addressed respondent general knowledge and attitudes regarding CPS‐GT decision‐making, focusing on the specific areas of (1) knowledge, interest, and parent versus child roles, with 13 items using a Likert response scale of 1 (strongly disagree) to 5 (strongly agree), (2) positive influencing factors for CPS‐GT decision‐making, with 10 items using a response scale of 1 (least important) to 5 (most important), and (3) negative influencing factors for CPS‐GT decision‐making, with 8 items using a response scale of 1 (least important) to 5 (most important).

### 2.4 Data analysis

The present analyses included baseline survey data from all parents and from patients within parent‐patient dyads. Descriptive statistics were calculated to examine demographic characteristics and knowledge and attitudes about CPS‐GT. For descriptive statistics, response frequencies were reported. Analyses of multicollinearity indicated high variance inflation factor (VIF >2.5) for the majority of sociodemographic factors.^23^ Therefore, independent samples *t*‐tests were used to examine sociodemographic group differences for CPS‐GT knowledge and attitudes. Given the pilot nature of the study, adjustments for Type I errors such as Bonferroni corrections were not used to prevent the risk of over‐correction.^24^ Further, research has demonstrated that parametric tests can provide more robust findings than non‐parametric tests during analysis of ordinal data; therefore, *t*‐tests were considered an appropriate type of analysis to utilize for this study.^25^, ^26^ Levene's test of heterogeneity of variance was also conducted, and Welch's *t*‐tests were conducted and reported if heterogeneity of variance was indicated. Welch's *t*‐tests are robust to Type I error and correct for unequal variances.^27^ All analyses were conducted using SPSS 28.0.

Parents were grouped based on the following demographic characteristics: race (White or non‐White aligning with literature suggesting health disparities between White and non‐White patients in GT and suggesting a need for further investigation of this in the literature^17^), ethnicity (Latino/Hispanic or non‐Latino/Hispanic), gender (male or female), employment status (employed or unemployed), education level (below or above 4‐year university education), income (<$75 000 or ≥$75 000 [median value in sample and value best aligned with median household income of $84 097 from 2017 to 2021, per US Census Bureau^28^]), language spoken at home (English or Spanish), language spoken in a medical setting (English or Spanish), and insurance (Medicaid or private insurance). These demographic factors represent measures of race, ethnicity, and socioeconomic status, which are reported as subject to health disparities in the literature.

## 3 RESULTS

### 3.1 Sociodemographic characteristics

See Table 1 for sociodemographic characteristics. Of the 105 parents who were approached, 94 agreed to participate and successfully completed baseline surveys; 19 of these were parents of patients between the ages of 15 and 18 who also completed baseline surveys. The mean parent age was 41.9 (SD = 8.3) and the mean patient age was 12.1 (SD = 5.5). For the parents, 77.7% were women, 78.5% identified as White, and 43.6% reported Latino/Hispanic ethnicity. Furthermore, 54.3% were employed, 47.8% had private health insurance, 40.4% had at least a four‐year university degree, 76.1% spoke English at home and 83.9% spoke English in a medical setting.

**TABLE 1:** Parent demographic characteristics.

Parent characteristic	*N* = 94 unless otherwise stated
Age (*N* = 83)	41.9 (8.3)
Gender	
Female (%)	73 (77.7)
Male (%)	21 (22.3)
Race (*N* = 93)	
White (%)	73 (78.5)
Black/African American (%)	3 (3.2)
Asian	6 (6.5)
Native Hawaiian or Pacific Islander	1 (1.1)
Other*	10 (10.8)
Ethnicity	
Latino/Hispanic (%)	41 (43.6)
Non‐Latino/Hispanic (%)	53 (56.4)
Income (*N* = 86)	
<$75 000	43 (50.0)
≥$75 000	43 (50.0)
Employment	
Employed (%)	51 (54.3)
Not Employed (%)	43 (45.7)
Language at Home (*N* = 92)	
English (%)	70 (76.1)
Spanish (%)	22 (23.9)
Language in Medical Setting (*N* = 93)	
English (%)	78 (83.9)
Spanish (%)	15 (16.1)
Child's age	12.1 (5.5)
Child's type of cancer (*N* = 93)	
Leukemia	39 (41.9)
Lymphoma	6 (6.5)
Bone Tumor	4 (4.3)
Brain or Spinal Cord Tumor	13 (14.0)
Neuroblastoma	6 (6.5)
Liver Tumor	1 (1.1)
Kidney Tumor	6 (6.5)
Soft Tissue Tumor or Muscle Tumor	6 (6.5)
Germ Cell Tumor or Tumor of the Testes or Ovaries	2 (2.2)
Skin Tumor	1 (1.1)
Other	7 (7.5)
Self‐reported as Unknown	2 (2.2)
Health Insurance (*N* = 90)	
Private Insurance	43 (47.8)
Medicaid	47 (52.2)
Education	
Less than Four‐year University (%)	56 (59.6)
Greater than Four‐year University (%)	38 (40.4)

* 7 identified as Latino/Hispanic, 1 identified as Chaldian, 1 identified as mixed race, and 1 did not disclose race.

### 3.2 Self‐reported parent knowledge and attitudes about CPS‐GT

See Table 2 for frequencies of parent responses. Regarding knowledge of CPS‐GT, most parents (over 86%) agreed/strongly agreed that they were aware that CPS‐GT could be used to diagnose cancer predispositions, that they understood what CPS‐GT was, that they were in favor of CPS‐GT for children, or that they were in favor of CPS‐GT for themselves or their children. About three quarters of parents agreed/strongly agreed with CPS‐GT even in the absence of prevention, treatment, or cure for the CPS, or that the benefits of CPS‐GT outweighed the risks. Regarding parent versus child decision‐making, most parents (89%) agreed/strongly agreed that parents should decide if children under age 18 should receive CPS‐GT, and 71% of parents agreed/strongly agreed that teenagers over 13 should be involved in CPS‐GT decision‐making (i.e., whether to receive CPS‐GT). Conversely, about a quarter of parents (66%) neither agreed nor disagreed and about 40% of parents disagreed/strongly disagreed that children aged 7–13 should be involved in CPS‐GT decision‐making. In terms of disclosure of CPS‐GT findings, the majority of parents (62%) agreed/strongly agreed that children and adolescents under 18 should be told immediately if they tested positive, while more parents (83%) agreed/strongly agreed that children and adolescents should be told immediately if they tested negative. Most parents (86%) agreed/strongly agreed that pediatricians should be informed of the results if their children tested positive.

**TABLE 2:** Parent knowledge and attitudes regarding CPS‐GT.

Question	N(%)	N(%)	N(%)	N(%)	N(%)
Knowledge, Interest, and Parent Versus Child Roles					
	Strongly Disagree	Disagree	Neither Agree nor Disagree	Agree	Strongly Agree
I am aware that genetic testing can be used to diagnose cancer predispositions (*N* = 91)	3 (3.3)	0 (0)	4 (4.4)	27 (29.7)	57 (62.6)
I understand what cancer predisposition genetic testing is (*N* = 92)	1 (1.1)	5 (5.4)	6 (6.5)	34 (37.0)	46 (50.0)
I am in favor of cancer predisposition genetic testing for children (9 = 92)	1 (1.1)	0 (0.0)	8 (8.7)	27 (29.3)	56 (60.9)
I am in favor of cancer predisposition genetic testing for children, even when there is no prevention, treatment or cure for the cancer predisposition (*N* = 92)	2 (2.2)	0 (0)	21 (22.8)	24 (26.1)	45 (48.9)
I am interested in taking a cancer predisposition genetic test for myself (*N* = 92)	1 (1.1)	1 (1.1)	10 (10.9)	30 (32.6)	50 (54.3)
I am interested in having my child take a cancer predisposition genetic test (*N* = 92)	1 (1.1)	0 (0)	5 (5.4)	32 (34.8)	54 (58.7)
The benefits of cancer predisposition genetic testing outweigh the risks (*N* = 92)	2 (2.2)	1 (1.1)	18 (19.6)	28 (30.4)	43 (46.7)
Parents should decide if children and adolescents less than 18 years of age should be tested for cancer predispositions (*N* = 93)	1 (1.1)	1 (1.1)	8 (8.6)	33 (35.5)	50 (53.8)
Teenagers above the age of 13 (thirteen) should be involved in making decisions for cancer predisposition genetic testing and communication of results (*N* = 93)	5 (5.4)	8 (8.6)	14 (15.1)	36 (38.7)	30 (32.3)
Children aged 7–13 (seven to thirteen) should be involved in making the decision for cancer predisposition genetic testing and communication of results (*N* = 93)	10 (10.8)	26 (28.0)	25 (26.9)	17 (18.3)	15 (16.1)
If children and adolescents less than 18 years of age are tested and they turn out to carry a cancer predisposition, they should be told about the test results immediately (*N* = 93)	1 (1.1)	11 (11.8)	23 (24.7)	23 (24.7)	35 (37.6)
If children and adolescents less than 18 years of age are tested and they turn out not to carry a cancer predisposition, they should be told about the test results immediately (*N* = 93)	0 (0)	7 (7.5)	9 (9.7)	29 (31.2)	48 (51.6)
Pediatricians of tested children and adolescents less than 18 years of age who turn out to carry a cancer predisposition should be told about the results (*N* = 93)	2 (2.2)	5 (5.4)	6 (6.5)	24 (25.8)	56 (60.2)

Positive Influencing Factors for CPS‐GT Decision‐Making					
	Least Important	Not Important	Neutral	Important	Most Important
Number of family members with cancer (*N* = 92)	4 (4.3)	4 (4.3)	12 (13.0)	42 (45.7)	30 (32.6)
Early age of cancer diagnosis in family members (*N* = 93)	2 (2.2)	3 (3.2)	9 (9.7)	35 (37.6)	44 (47.3)
Presence of known cancer predisposition syndrome in family member (*N* = 94)	4 (4.3)	3 (3.2)	11 (11.7)	31 (33.0)	45 (47.9)
Your child's age at initial cancer diagnosis (*N* = 92)	1 (1.1)	1 (1.1)	9 (9.8)	44 (47.8)	37 (40.2)
Your child's doctors recommendation for cancer predisposition genetic testing (*N* = 93)	1 (1.1)	3 (3.2)	16 (17.2)	43 (46.2)	30 (32.3)
Distinctive (special or particular) characteristics of your childs cancer (*N* = 94)	1 (1.1)	1 (1.1)	10 (10.6)	38 (40.4)	44 (46.8)
Potential impact on further treatment for your child (*N* = 94)	0 (0)	0 (0)	5 (5.3)	26 (27.7)	63 (67.0)
Potential impact on future surveillance for your child (*N* = 92)	0 (0)	0 (0)	5 (5.4)	24 (26.1)	63 (68.5)
It is important to have as much information as possible about cancer predispositions for your child's future (*N* = 93)	1 (1.1)	1 (1.1)	3 (3.2)	17 (18.3)	71 (76.3)
I want to know why my child has developed cancer, regardless of practical impact (*N* = 93)	1 (1.1)	4 (4.3)	12 (12.9)	22 (23.7)	54 (58.1)

Negative Influencing Factors for CPS‐GT Decision‐Making					
	Least Important	Not Important	Neutral	Important	Most Important
Cancer predisposition genetic testing is not helpful (*N* = 91)	37 (40.7)	7 (7.7)	13 (14.3)	19 (20.9)	15 (16.5)
Cancer predisposition genetic testing may cause psychological distress to me (*N* = 93)	18 (19.4)	13 (14.0)	24 (25.8)	24 (25.8)	14 (15.1)
Cancer predisposition genetic testing may cause psychological distress to my child (*N* = 94)	8 (8.5)	7 (7.4)	26 (27.7)	29 (30.9)	24 (25.5)
Cancer predisposition genetic testing may cause psychological distress to the patients family (*N* = 93)	9 (9.7)	9 (9.7)	26 (28.0)	28 (30.1)	21 (22.6)
Patient confidentiality/privacy will be at risk (*N* = 92)	13 (14.1)	11 (12.0)	34 (37.0)	17 (18.5)	17 (18.5)
Cancer predisposition genetic testing is too costly (*N* = 94)	18 (19.1)	5 (5.3)	36 (38.3)	17 (18.1)	18 (19.1)
Patients with positive test results may face discrimination at work or when seeking insurance (*N* = 94)	13 (13.8)	9 (9.6)	22 (23.4)	29 (30.9)	21 (22.3)
A positive result in cancer predisposition genetic testing may negatively affect relationships with patients family and relatives (*N* = 94)	20 (21.3)	18 (19.1)	25 (26.6)	20 (21.3)	11 (11.7)

Regarding positive influencing factors for CPS‐GT decision‐making (i.e., whether to receive CPS‐GT), almost every parent (95%) rated having as much information as possible about CPS for their child's future, potential impact on future surveillance for their child, or potential impact on further treatment for their child as important/most important in influencing CPS‐GT decision‐making. A high percentage of parents (82%) also rated knowing why their child developed cancer, regardless of practical impact, as important/most important. Regarding other influencing factors for CPS‐GT decision‐making, between 80% and 90% of parents rated distinctive characteristics of their child's cancer, their child's doctor's recommendation, their child's age at initial diagnosis, or early age of cancer diagnosis in family members as important/most important. Additionally, about 80% of parents rated the presence of known CPS in a family member or number of family members with cancer as important/most important.

Regarding negative influencing factors for CPS‐GT decision‐making (i.e., whether to receive CPS‐GT), about half of parents rated CPS‐GT not being helpful as an unimportant/least important influencing factor for CPS‐GT decision‐making, while 37% of parents rated it as an important/most important and 14.3% rated it as a neutral influencing factor. While 40.9% of parents rated psychological distress to oneself as an important/most important influencing factor, a quarter of parents rated it as neutral and 33% of parents rated it as unimportant/least important. The majority of parents (52% to 56%) rated psychological distress to one's child or psychological distress to one's family as important/most important concerns. Equal percentages of parents (37%) rated patient confidentiality/privacy being at risk and CPS‐GT costliness as important/most important influencing factors, while a similar number (37%) of parents rated these as neutral influencing factors. About half of parents rated workplace or insurance‐based discrimination as a result of a positive test as an important/most important concern. Finally, 33% of parents rated the negative impact of a positive result on family relationships as an important/most important influencing factor, while about a quarter of parents rated it as a neutral influencing factor and 40% rated it as an unimportant/least important influencing factor.

### 3.3 Self‐reported patient knowledge and attitudes regarding CPS‐GT decision‐making

See Table 3 for a frequency table of patient responses (*n* = 19). Regarding knowledge of CPS‐GT, almost all patients (95%) agreed/strongly agreed that they were aware that CPS‐GT could be used to diagnose cancer predispositions and 68% agreed/strongly agreed that they understood what CPS‐GT was. Regarding interest in CPS‐GT, about 80% of patients agreed/strongly agreed that they were in favor of CPS‐GT for children or for themselves specifically; however, only about half of patients agreed/strongly agreed with CPS‐GT even in the absence of prevention, treatment, or cure for the CPS, or that the benefits of CPS‐GT outweighed the risks. Regarding parent versus child decision‐making, about half of patients agreed/strongly agreed, 32% felt neutral, and 21% disagreed/strongly disagreed that parents should decide if children and adolescents under 18 should be tested for CPS. Most patients (more than 84%) agreed/strongly agreed that teenagers over 13 should be involved in CPS‐GT decision‐making or that children and adolescents should be told immediately if they tested positive or if they tested negative. The same number of patients agreed/strongly agreed or felt neutral (42%) regarding whether children aged 7–13 should be involved in CPS‐GT decision‐making, while 16% disagreed/strongly disagreed. Most patients (95%) agreed/strongly agreed that pediatricians should be informed of the results if they tested positive.

**TABLE 3:** Patient knowledge and attitudes regarding CPS‐GT.

Question	*N*(%)	*N*(%)	*N*(%)	*N*(%)	*N*(%)
Knowledge, Interest, and Parent Versus Child Roles					
	Strongly Disagree	Disagree	Neither Agree nor Disagree	Agree	Strongly Agree
I am aware that genetic testing can be used to diagnose cancer predispositions (*N* = 19)	0 (0)	0 (0)	1 (5.3)	13 (68.4)	5 (26.3)
I understand what cancer predisposition genetic testing is (*N* = 19)	0 (0)	2 (10.5)	4 (21.1)	10 (52.6)	3 (15.8)
I am in favor of cancer predisposition genetic testing for children (*N* = 19)	0 (0)	0 (0)	4 (21.1)	11 (57.9)	4 (21.1)
I am in favor of cancer predisposition genetic testing for children, even when there is no prevention, treatment or cure for the cancer predisposition (*N* = 19)	1 (5.3)	0 (0)	8 (42.1)	7 (36.8)	3 (15.8)
I am interested in taking a cancer predisposition genetic test for myself (*N* = 19)	0 (0)	0 (0)	4 (21.1)	12 (63.2)	3 (15.8)
The benefits of cancer predisposition genetic testing outweigh the risks (*N* = 19)	0 (0)	1 (5.3)	8 (42.1)	7 (36.8)	3 (15.8)
Parents should decide if children and adolescents less than 18 years of age should be tested for cancer predispositions (*N* = 19)	1 (5.3)	3 (15.8)	6 (31.6)	6 (31.6)	3 (15.8)
Teenagers above the age of 13 (thirteen) should be involved in making decisions for cancer predisposition genetic testing and communication of results (*N* = 19)	0 (0)	0 (0)	4 (21.1)	10 (52.6)	5 (26.3)
Children aged 7–13 (seven to thirteen) should be involved in making the decision for cancer predisposition genetic testing and communication of results (*N* = 19)	0 (0)	3 (15.8)	8 (42.1)	5 (26.3)	3 (15.8)
If children and adolescents less than 18 years of age are tested and they turn out to carry a cancer predisposition, they should be told about the test results immediately (*N* = 19)	0 (0)	0 (0)	3 (15.8)	8 (42.1)	8 (42.1)
If children and adolescents less than 18 years of age are tested and they turn out not to carry a cancer predisposition, they should be told about the test results immediately (*N* = 18)	0 (0)	0 (0)	2 (11.1)	6 (33.3)	10 (55.6)
Pediatricians of tested children and adolescents less than 18 years of age who turn out to carry a cancer predisposition should be told about the results (*N* = 19)	0 (0)	0 (0)	1 (5.3)	8 (42.1)	10 (52.6)

Positive Influencing Factors for CPS‐GT Decision‐Making					
	Least Important	Not Important	Neutral	Important	Most Important
Number of family members with cancer (*N* = 19)	1 (5.3)	0 (0)	2 (10.5)	11 (57.9)	5 (26.3)
Early age of cancer diagnosis in family members (*N* = 19)	1 (5.3)	0 (0)	3 (15.8)	12 (63.2)	3 (15.8)
Presence of known cancer predisposition syndrome in family member (*N* = 19)	1 (5.3)	0 (0)	3 (15.8)	9 (47.4)	6 (31.6)
Your age at initial cancer diagnosis (*N* = 19)	1 (5.3)	1 (5.3)	1 (5.3)	14 (73.7)	2 (10.5)
Your doctor's recommendation for cancer predisposition genetic testing (*N* = 19)	1 (5.3)	0 (0)	7 (36.8)	9 (47.4)	2 (10.5)
Distinctive (special or particular) characteristics of your childs cancer (*N* = 19)	1 (5.3)	0 (0)	2 (10.5)	12 (63.2)	4 (21.1)
Potential impact on further treatment for your child (*N* = 19)	1 (5.3)	0 (0)	3 (15.8)	8 (42.1)	7 (36.8)
Potential impact on future surveillance for your child (*N* = 18)	0 (0)	0 (0)	3 (15.8)	11 (57.9)	5 (26.3)

Negative Influencing Factors for CPS‐GT Decision‐Making					
	Least Important	Not Important	Neutral	Important	Most Important
Cancer predisposition genetic testing is not helpful (*N* = 19)	6 (31.6)	4 (21.1)	5 (26.3)	4 (21.1)	0 (0)
Cancer predisposition genetic testing may cause psychological distress to me (*N* = 18)	2 (10.5)	4 (21.1)	7 (36.8)	5 (26.3)	1 (5.3)
Cancer predisposition genetic testing may cause psychological distress to the patient's family (*N* = 19)	0 (0)	5 (26.3)	6 (31.6)	7 (36.8)	1 (5.3)
Patient confidentiality/privacy will be at risk (*N* = 19)	1 (5.3)	4 (21.1)	6 (31.6)	6 (31.6)	2 (10.5)
Cancer predisposition genetic testing is too costly (*N* = 19)	1 (5.3)	2 (10.5)	9 (47.4)	7 (36.8)	0 (0)
Patients with positive test results may face discrimination at work or when seeking insurance (*N* = 19)	1 (5.6)	3 (16.7)	6 (33.3)	6 (33.3)	2 (11.1)
A positive result in cancer predisposition genetic testing may negatively affect relationships with patients family and relatives (*N* = 19)	2 (10.5)	6 (31.6)	5 (26.3)	5 (26.3)	1 (5.3)

Regarding positive influencing factors for CPS‐GT decision‐making, most patients (78% to 84%) rated potential impact on future surveillance, potential impact on future treatment, and distinctive characteristics of their cancer as important/most important in influencing CPS‐GT decision‐making. Similarly, most patients (78% to 84%) rated their age at initial diagnosis, early age of cancer diagnosis in family members, known CPS in a family member, and number of family members with cancer as important/most important influencing factors. A little over half of patients rated their doctor's recommendation for CPS‐GT as an important/most important influencing factor.

Regarding negative influencing factors for CPS‐GT decision‐making, about half of patients rated CPS‐GT not being helpful as an unimportant/least important influencing factor in CPS‐GT decision making. The same number of patients (32%) rated psychological distress to oneself was rated as an important/most important or unimportant/least important influencing factor. In addition, 42% of patients rated psychological distress to one's family as an unimportant/least important, while 32% rated it as an important/most important influencing factor. Slightly less than half of patients (42% to 44%) rated patient confidentiality/privacy being at risk or workplace or insurance‐based discrimination as a result of a positive test as important/most important influencing factors, while 32% to 33% of patients rated these considerations as neutral influencing factors. Almost half of patients (47%) rated CPS‐GT costliness as a neutral influencing factor, while 37% rated it as an important/most important influencing factor.

### 3.4 Sociodemographic differences in parent knowledge and attitudes regarding CPS‐GT decision‐making

See Supplemental Tables S1–S4 for all means and statistical findings regarding CPS‐GT‐related knowledge and attitudes. Statistically significant findings are described here.

#### 3.4.1 Race and ethnicity

There were no significant differences by race (White versus non‐White, i.e., Black or Other). For ethnicity, Latino/Hispanic parents believed more strongly than non‐Latino/Hispanic parents that children under 18 should be told immediately about CPS‐GT findings, whether children carried a genetic predisposition or not. Further, Latino/Hispanic parents felt more strongly than non‐Latino/Hispanic parents that CPS‐GT not being helpful was an important factor in CPS‐GT decision‐making, as well as CPS‐GT costliness. Finally, Latino/Hispanic parents felt more strongly than non‐Latino/Hispanic parents that having as much information as possible about CPS for their child's future was an important factor in CPS‐GT decision‐making.

#### 3.4.2 Gender

Male parents believed more strongly than female parents that the presence of a known CPS in family members would make them more likely to participate in CPS‐GT. Female parents believed more strongly that the potential personal psychological distress related to CPS‐GT was an important factor in CPS‐GT decision‐making.

#### 3.4.3 Language spoken at home

Parents who spoke Spanish at home were more likely than parents who spoke English at home to want to know why their child developed cancer, regardless of practical impact, and to value having as much information as possible about cancer predispositions for their child's future, as influencing factors for CPS‐GT decision‐making. Parents who spoke Spanish at home were also more likely to believe that CPS‐GT not being helpful was an important factor in CPS‐GT decision‐making, in addition to the personal psychological distress it could cause.

#### 3.4.4 Language spoken in a medical setting

Parents who spoke Spanish in a medical setting were more likely than parents who spoke English in a medical setting to believe that children ages 7 to 13 should be involved in making the decision for CPS‐GT and communication of results and that children under 18 should be told about testing results if they carry a CPS. They were more likely to believe that pediatricians of minors with CPS should be told about the results. They were also more likely to want to know why their child developed cancer, regardless of practical impact, and to value having as much information as possible about cancer predispositions for their child's future. They believed more strongly that CPS‐GT not being helpful was an important factor in CPS‐GT decision‐making in addition to its costliness. Additionally, they believed more strongly that the potential personal and familial psychological distress related to CPS‐GT was an important factor in CPS‐GT decision‐making. Parents who spoke English in a medical setting were more likely to be influenced by the presence of known CPS in a family member in CPS‐GT decision‐making.

#### 3.4.5 Income

Parents with a household income less than $75 000 were more likely than parents with a household income $75 000 or greater to believe that children under 18 should be told about testing results if they carried a CPS and that CPS‐GT not being helpful was an important factor in CPS‐GT decision‐making.

#### 3.4.6 Insurance

Parents with Medicaid believed more strongly than parents with private insurance that children under 18 should be told about testing results, whether they carried a CPS or not, and that the potential impact on their child's future surveillance would influence their CPS‐GT decision‐making. Furthermore, parents with Medicaid were more likely to believe that CPS‐GT not being helpful and potential psychological distress related to CPS‐GT were important factors in CPS‐GT decision‐making.

#### 3.4.7 Employment

Unemployed parents believed more strongly than employed parents that children ages 7–13 should be involved in decision‐making for CPS‐GT and communication of results, and that children and adolescents under 18 should be told about the results whether they carried a genetic predisposition or not.

#### 3.4.8 Education

Parents without a four‐year university degree indicated less awareness that CPS‐GT could be used to diagnose CPS than parents with a four‐year university degree. Parents without a four‐year university degree were more interested in taking a CPS‐GT for themselves than parents with at least a four‐year university degree and believed more strongly that children and adolescents under age 18 should be told about test results if they carried a CPS. They were also more likely than parents with at least four years of university education to believe that CPS‐GT not being helpful and too costly were important factors in CPS‐GT decision‐making.

### 3.5 Parent versus patient knowledge and attitudes regarding CPS‐GT decision‐making

See Supplementary Material B for all means and statistical findings for responses regarding CPS‐GT‐related knowledge and attitudes. Among the 19 parent‐patient dyads, parents believed more strongly than their children that they understood what CPS‐GT was and had a higher preference for CPS‐GT for children under 18 even if prevention, treatment, or a cure wasn't available. Further, parents believed more strongly that the benefits of CPS‐GT outweighed the risks, and that parents should decide if children under 18 should be tested for CPS. Parents also believed more strongly that the potential impact on further treatment for the patient was important in GT decision‐making. There were no significant parent‐patient differences on other knowledge and attitude items.

## 4 DISCUSSION

Parents largely demonstrated a strong motivation to participate in CPS‐GT and agreed that the benefits outweighed the risks, aligning with previous literature.^1^, ^8^ Parents, regardless of sociodemographic background, agreed that parents should make decisions about CPS‐GT and that patients over the age of 13 should be included in these decisions.

Parental sociodemographic factors were associated with perceptions of CPS‐GT decision making and risk assessment, although the small sample size indicates the need for more highly powered studies investigating these considerations. Latino/Hispanic parents and parents who spoke Spanish in medical settings believed more strongly that their children should be told about CPS‐GT results, and parents who spoke Spanish in medical settings believed more strongly that their children should be involved in GT decision‐making. Parents who spoke Spanish in medical settings also had greater concerns about the utility of CPS‐GT and the potential distress associated with it, and had lower understanding about influencing factors for proceeding with CPS‐GT, such as known family members with CPS. Study findings suggest that these parents may need additional support in understanding CPS‐GT implications.^17^

Previous literature has not differentiated between English and Spanish‐speaking individual knowledge and attitudes regarding CPS‐GT decision‐making, but has indicated that Latino/Hispanic individuals may have less factual knowledge regarding GT and that patients from racial/ethnic minority backgrounds have higher physician distrust in medical settings.^29^ Translation alone is likely not sufficient in medical settings, and cultural considerations should be simultaneously taken when delivering medical care.^30^ Providers may be inclined to assume similar preferences for all Latino/Hispanic patients and parents, but unique considerations should be based on a broader set of characteristics, including personal medical language preferences.

There were also notable associations of attitudes of parents with lower socioeconomic status (i.e., unemployed, with incomes lower than $75 000, less than four‐year university education, Medicaid insurance). These parents were more inclined to believe that CPS‐GT may not be helpful and that it may be too costly, aligning with previous findings,^1^ and also had stronger preferences for patient autonomy in CPS‐GT decision‐making. The present study removed the cost barrier to CPS‐GT by offering free counseling and optional testing, but skepticism and concern over the consequences of CPS‐GT remained. These findings suggest that even when there is no cost burden, it is still important for providers to have conversations with patients that mitigate their concerns. Having a genetic counselor provide information on procedures and implications can alleviate concerns and strengthen familial understanding of CPS‐GT implications, making genetic counselors important resources to mitigate challenges beyond cost burden.^7^ It is also important for medical providers, especially genetic counselors, to build trust through clearly conveying CPS‐GT procedures and implications.^7^, ^12^

Parents and patients were in alignment on a variety of factors regarding CPS‐GT decision‐making, including high interest in CPS‐GT, agreeing that patients should be told about results, and agreeing that teenagers should be involved in CPS‐GT decision‐making. Parents felt more strongly that the benefits outweighed the costs of CPS‐GT, that CPS‐GT should be pursued regardless of practical implications, and that parents should make CPS‐GT decisions. Parents also felt more strongly that future treatment and surveillance options should influence CPS‐GT decision‐making. Previous literature suggests that parents and patients differ in opinions on decisional autonomy for GT and that patients may experience distress when CPS‐GT results are shared by parents through unplanned conversations.^10^ Importantly, our study only included parents and patients post‐cancer diagnosis, who face frequent high‐stakes medical decisions. Patients in this study expressed that they desired to have a role in the CPS‐GT decision‐making process, and this should be taken into account, especially when adolescent patients are getting closer to the age of 18 when they'll gain more medical decision‐making autonomy. For example, if CPS‐GT lacks practical implications, it is possible that genetic counselors may have increased challenges balancing parent and patient preferences. Study findings support the importance of a collaborative parent–child approach in navigating the CPS‐GT decision‐making process, especially for teenagers with cancer.^8^, ^31^ Given that the sample size for dyads (19 parents and 19 patients) was small due to the pilot nature of the study, future studies should continue this research with larger sample sizes that can also account for sociodemographic differences in both parents and patients, and how these sociodemographic differences may contribute to differences in parent and patient knowledge and attitudes regarding CPS‐GT decision‐making.

Findings can be considered alongside the unique ethical issues presented by pediatric GT. Concerns of stigma, discrimination and misinterpretation of results continue to impact the standardized implementation of GT in pediatric populations.^32^ Notably, a third of parents in our sample reported concerns that patient confidentiality/privacy being at risk. Half of parents were concerned about workplace or insurance‐based discrimination as a result of a positive test. Parents from different sociodemographic backgrounds, and parents and children, were aligned on these issues. Helping parents and patients consider the implications of CPS‐GT should be incorporated into collaborative parent and child CPS‐GT decision‐making processes.

Findings should also be considered within the context of broader healthcare disparities for underrepresented minority populations. Patients from underrepresented minority backgrounds experience higher rates of language barriers, health literacy challenges, accessibility limitations, and discrimination and stigmatization in healthcare settings.^33^ They are also vulnerable to higher rates of cancer‐related morbidity and mortality. In the fields of genetic counseling and genomic medicine, studies report a lack of sociodemographic diversity among genetic counseling providers, biases and blind spots in communication, and lack of representation in genomic research. The National Society of Genetic Counselors has proposed introducing genetic counseling as a profession earlier in educational settings, particularly to promote multilingual engagement in the profession, genetic counseling‐specific cultural competency training, and introducing more accessible genetic counseling‐specific community clinics, particularly targeting culturally‐specific genetic mutations.^34^ These steps could potentially open the field to greater health equity for underrepresented minority populations.

This study assessed factors influencing CPS‐GT decision‐making, patient involvement in decision‐making, and consequences, which have not been collectively explored in previous studies. Furthermore, this study's incorporation of patient knowledge and attitudes regarding CPS‐GT decision‐making is a strength, given the minimal literature in this area. Limitations include the small number of patients in this study, as only patients between ages 15 and 18 completed surveys. Further, a study‐specific assessment was used and while it drew from validated and reliable surveys on adult CPS‐GT, it was not piloted in adolescents prior to use. These considerations, along with the local sample and low power, limited the generalizability of findings. In addition, analyses did not include objective measures of CPS‐GT‐related knowledge, such as assessments of health literacy, and perceived knowledge versus objective knowledge can differ.^35^ Although the sample had substantial Latino/Hispanic representation, racial diversity was more limited. Finally, all participants had agreed to participate in the study knowing that genetic counseling would be offered (with the option for CPS‐GT), thereby potentially biasing the sample toward those who are open to CPS‐GT. Future studies should be directed toward assessing viewpoints of those who may not be inclined to pursue CPS‐GT.

This study provided important insights on sociodemographic and parent versus child similarities and differences in knowledge and attitudes regarding CPS‐GT decision‐making. Results indicate the importance of taking parent sociodemographic backgrounds into consideration when relaying information about CPS‐GT, especially for Spanish‐speaking parents who may face additional communication barriers. Further, clinicians should consider that parents and patients may differ in knowledge and attitudes regarding CPS‐GT decision‐making. Study findings suggest a need for larger scale studies investigating potential differences in sociodemographic and parent versus child knowledge and attitudes regarding CPS‐GT decision‐making. In the future, it may be valuable to develop interventions to close information gaps and addressing diverse parent and patient concerns regarding CPS‐GT.

## AUTHOR CONTRIBUTIONS

CSR contributed to formal analysis, writing—original draft, writing—review and editing, and visualization. DMF contributed to conceptualization, methodology, writing—review and editing, and project administration. SM contributed to methodology and project administration. AKC contributed to formal analysis, writing—original draft, writing—review and editing. SO contributed to formal analysis, writing—original draft, writing—review and editing. MK contributed to formal analysis, writing—original draft, writing—review and editing. VH contributed to conceptualization, methodology, project administration, writing—original draft, writing—review and editing, and project administration. DD contributed to conceptualization, methodology, project administration, and writing—review and editing. VLM contributed to formal analysis, writing—original draft, writing—review and editing. DJK contributed to conceptualization, methodology, project administration, writing—review and editing, and project administration.

## CONFLICT OF INTEREST STATEMENT

David Dimmock is employed by Creyon Bio, Inc. He reports previous consulting fees from Audentes and BioMarin Pioneering Medicine VII, Inc. He serves on a scientific advisory board for Taysha Gene Therapies and is an inventor on US patent 8718950B2 assigned to The Hudson Alpha Institute for Biotechnology.

## ETHICS STATEMENT

This study was approved by UCSD's Human Research Protections Program (UCSD IRB #181972). This study was performed in accordance with the Declaration of Helsinki.

## Supporting information


**Data S1:** Supplementary Information.

## Data Availability

The data that support the findings of this study are available on request from the corresponding author. The data are not publicly available due to privacy or ethical restrictions.
