# Supplementary material for: Parent and patient knowledge and attitudes about cancer predisposition syndrome genetic testing in pediatric oncology: Understanding sociodemographic and parent–child differences
Source: Cancer Rep (Hoboken). 2024 Sep 5;7(9):e2119. doi: 10.1002/cnr2.2119 (PMC11375323; doi:10.1002/cnr2.2119)
Supplement: Supplementary file 1 — Data S1: Supplementary Information. [file CNR2-7-e2119-s001.pdf]

Supplemental Table S1. Means and Statistical Findings for Race, Ethnicity, and Gender (bolded text indicates significant findings with $p < .05$)

Question	Race				Ethnicity				Gender			
	White (N = 73) Mean	Non-White (N = 20) Mean	T-value	P-value	Non-Hispanic (N = 53) Mean	Hispanic (N = 41) Mean	T-value	P-value	Male (N = 21) Mean	Female (N = 73) Mean	T-value	P-value
Knowledge, Interest, and Parent Versus Child Roles												
I am aware that genetic testing can be used to diagnose cancer predispositions	4.51(.79)	4.35(1.09)	.40*	.54	4.63(.56)	4.30(1.11)	1.70*	.10*	4.52(.75)	4.47(.90)	.24	.81
I understand what cancer predisposition genetic testing is	4.31(.89)	4.20(.95)	.48	.63	4.29(.86)	4.329(.96)	.01	.99	4.24(.94)	4.31(.89)	-.32	.75
I am in favor of cancer predisposition genetic testing for children and adolescents less than 18 years of age	4.48(.77)	4.50(.69)	-.12	.91	4.47(.64)	4.51(.87)	-.26	.79	4.62(.67)	4.45(.77)	.90	.37
I am in favor of cancer predisposition genetic testing for children, even when there is no prevention, treatment or cure for the cancer predisposition	4.14(.98)	4.35(.81)	-.88	.38	4.06(.90)	4.37(.97)	-1.57	.12	4.38(.81)	4.14(.98)	1.03	.31
I am interested in taking a cancer predisposition genetic test for myself	4.35(.81)	4.45(.76)	-.47	.64	4.25(.80)	4.54(.81)	-1.68	.10	4.57(.51)	4.32(.88)	1.63*	.11*
I am interested in having my child take a cancer predisposition genetic test	4.46(.73)	4.60(.60)	-.76	.45	4.47(.61)	4.554(.81)	-.45	.66	4.52(.60)	4.49(.73)	.18	.86
The benefits of cancer predisposition genetic testing outweigh the risks	4.14(1.00)	4.30(.73)	-.67	.51	4.20(.78)	4.17(1.12)	.12*	.90*	4.33(.80)	4.14(.98)	.83	.41
Parents should decide if children and adolescents less than 18 years of age should be tested for cancer predispositions	4.38(.78)	4.45(.83)	-.38	.71	4.37(.69)	4.44(.90)	-.45	.66	4.52(.60)	4.36(.83)	.84	.41
Teenagers above the age of 13 (thirteen) should be involved in making decisions for cancer predisposition genetic testing and communication of results	3.82(1.13)	3.85(1.18)	-.11	.92	3.92(.97)	3.73(1.32)	.78*	.44*	3.62(1.20)	3.90(1.12)	-1.01	.32
Children aged 7-13 (seven to thirteen) should be involved in making the decision for cancer predisposition genetic testing and communication of results	3.04(1.22)	2.80(1.23)	.77	.44	2.85(1.09)	3.22(1.41)	-1.40*	.17*	2.71(1.35)	3.10(1.21)	-1.24	.11
If children and adolescents less than 18 years of age are tested and they turn out to carry a cancer predisposition, they should be told about the test results immediately	3.75(1.11)	4.20(.95)	-1.65	.10	**3.60(1.05)**	**4.20(1.05)**	**-2.72**	**.01**	4.05(1.07)	3.81(1.10)	.90	.86
If children and adolescents less than 18 years of age are tested and they turn out not to carry a cancer predisposition, they should be told about the test results immediately	4.24(.93)	4.35(.93)	-.49	.63	**4.08(.95)**	**4.51(.84)**	**-2.31**	**.02**	4.24(1.04)	4.28(.89)	-.17	.86
Pediatricians of tested children and adolescents less than 18 years of age who turn out to carry a cancer predisposition should be told about the results	4.42(.95)	4.15(1.09)	1.08	.28	4.21(1.09)	4.56(.78)	-1.73	.09	4.24(1.14)	4.40(.93)	-.68	.50
Positive Influencing Factors for CPS-GT Decision-Making												
Number of family members with cancer	3.94(1.03)	4.05(1.00)	-.41	.68	4.00(.93)	3.95(1.13)	.23*	.41*	4.00(1.14)	3.97(.99)	.11	.91
Early age of cancer diagnosis in family members	4.19(.96)	4.40(.75)	-.89	.38	4.23(.87)	4.23(.87)	-.25	.82	4.57(.75)	4.15(.94)	1.87	.07
Presence of known cancer predisposition syndrome in family member	4.11(1.06)	4.35(.99)	-.91	.37	4.21(1.01)	4.12(1.10)	.29	.70	**4.62(.59)**	**4.04(1.11)**	**2.29**	**.02**
Your childs age at initial cancer diagnosis	4.21(.77)	4.37(.76)	-.81	.42	4.23(.70)	4.29(.86)	-.34	.73	4.52(.51)	4.17(.81)	1.89	.06
Your childs doctors recommendation for cancer predisposition genetic testing	3.96(.88)	4.35(.67)	-1.85	.07	3.96(.85)	4.18(.84)	-1.29	.24	4.33(.73)	3.97(.87)	1.73	.09
Distinctive (special or particular) characteristics of your childs cancer	4.29(.83)	4.35(.67)	-.31	.76	4.30(.77)	4.32(.82)	-.09	.93	4.52(.68)	4.25(.81)	1.43	.16
Potential impact on further treatment for your child	4.63(.57)	4.55(.69)	.54	.59	4.62(.60)	4.62(.59)	.11	.92	4.71(.46)	4.59(.62)	.86	.39
Potential impact on future surveillance for your child	4.65(.56)	4.53(.70)	.83	.20	4.55(.64)	4.73(.50)	-1.53*	.13*	4.55(.61)	4.65(.59)	-.69	.49
It is important to have as much information as possible about cancer predispositions for your childs future	4.67(.73)	4.68(.58)	-.07	.94	**4.54(.85)**	**4.85(.36)**	**-2.41**	**.02**	4.85(.37)	4.63(.76)	1.83*	.07*
I want to know why my child has developed cancer, regardless of practical impact	4.29(1.01)	4.47(.61)	-1.02*	.32*	4.17(1.02)	4.54(.76)	-1.95*	.06*	4.45(.89)	4.30(.95)	.63	.53
Negative Influencing Factors for CPS-GT Decision-Making												
Cancer predisposition genetic testiang is not helpful	2.62(1.59)	2.76(1.60)	-.35	.73	**2.04(1.36)**	**3.43(1.50)**	**-4.62**	**<.001**	2.16(1.50)	2.78(1.58)	-1.54	.13
Cancer predisposition genetic testing may cause psychological distress to me	2.97(1.36)	3.26(1.28)	-.84	.41	2.85(1.27)	3.27(1.40)	-1.52	.13	**2.45(1.28)**	**3.19(1.32)**	**-2.24**	**.03**
Cancer predisposition genetic testing may cause psychological distress to my child	3.55(1.14)	3.60(1.39)	-.17	.86	3.51(1.20)	3.66(1.20)	-.60	.55	3.14(1.49)	3.70(1.10)	-1.90	.06
Cancer predisposition genetic testing may cause psychological distress to the patient's family	3.38(1.19)	3.68(1.34)	-.96	.34	3.36(1.21)	3.60(1.24)	-.94	.35	3.14(1.49)	3.56(1.12)	-1.37	.17
Patient confidentiality/privacy will be at risk	3.11(1.26)	3.32(1.34)	-.62	.54	3.04(1.28)	3.29(1.25)	-.95	.34	3.32(1.64)	3.11(1.16)	.52*	.61*
Cancer predisposition genetic testing is too costly	3.00(1.34)	3.60(1.23)	-1.89	.07	**2.87(1.39)**	**3.46(1.19)**	**-2.20**	**.03**	3.00(1.55)	3.16(1.27)	-.50	.62
Patients with positive test results may face discrimination at work or when seeking insurance	3.41(1.29)	3.30(1.46)	.10*	.76	3.32(1.22)	3.46(1.43)	-.52	.30	3.33(1.65)	3.40(1.21)	-.17*	.87
A positive result in cancer predisposition genetic testing may negatively affect relationships with patient's family and relatives	2.71(1.25)	3.25(1.48)	-1.63	.11	2.70(1.27)	3.00(1.36)	-.11	.14	2.86(1.53)	2.82(1.25)	.11	.91

* Adjusted for heterogeneity of variance using Welch's t-test

Supplementary Material S2. Means and Statistical Findings for Language at Home and Language in Medical Settings (bolded text indicates significant findings with $p < .05$)

Question	Language at Home				Language in a Medical Setting			
	English (N = 70) Mean	Spanish (N = 22) Mean	T-value	P-value	English (N = 78) Mean	Spanish (N = 15) Mean	T-value	P-value
Knowledge, Interest, and Parent Versus Child Roles								
I am aware that genetic testing can be used to diagnose cancer predispositions	4.54(.82)	4.33(1.02)	.97	.33	4.49(.90)	4.5(.65)	-.05	.96
I understand what cancer predisposition genetic testing is	4.32(.85)	4.23(1.02)	.44	.66	4.26(.96)	4.47(.52)	-.89	.43
I am in favor of cancer predisposition genetic testing for children and adolescents less than 18 years of age	4.49(.66)	4.55(.96)	-.33	.74	4.47(.52)	4.47(.76)	-.93	.36
I am in favor of cancer predisposition genetic testing for children, even when there is no prevention, treatment or cure for the cancer predisposition	4.13(.91)	4.45(1.01)	-1.40	.16	4.14(.96)	4.53(.74)	-1.48	.14
I am interested in taking a cancer predisposition genetic test for myself	4.35(.79)	4.45(.91)	-.51	.61	4.34(.86)	4.60(.51)	-1.12	.26
I am interested in having my child take a cancer predisposition genetic test	4.51(.61)	4.45(.96)	.35	.73	4.50(.72)	4.53(.64)	-.17	.87
The benefits of cancer predisposition genetic testing outweigh the risks	4.19(.89)	4.14(1.13)	.24	.81	4.22(.93)	4.07(.96)	.59	.56
Parents should decide if children and adolescents less than 18 years of age should be tested for cancer predispositions	4.39(.73)	4.41(.96)	-.09	.93	4.36(.81)	4.53(.64)	-.77	.45
Teenagers above the age of 13 (thirteen) should be involved in making decisions for cancer predisposition genetic testing and communication of results	3.86(1.12)	3.82(1.26)	.13	.90	3.74(1.16)	4.27(.88)	-1.66	.10
Children aged 7-13 (seven to thirteen) should be involved in making the decision for cancer predisposition genetic testing and communication of results	2.88(1.16)	3.45(1.57)	-1.66*	.11*	**2.82(1.18)**	**3.93(1.22)**	**-3.33**	**.001**
If children and adolescents less than 18 years of age are tested and they turn out to carry a cancer predisposition, they should be told about the test results immediately	3.75(1.09)	4.14(1.08)	-1.44	.15	**3.74(1.08)**	**4.40(.99)**	**-2.19**	**.03**
If children and adolescents less than 18 years of age are tested and they turn out not to carry a cancer predisposition, they should be told about the test results immediately	4.22(.91)	4.41(1.01)	-.84	.40	4.21(.92)	4.53(.92)	-1.25	.21
Pediatricians of tested children and adolescents less than 18 years of age who turn out to carry a cancer predisposition should be told about the results	4.36(.95)	4.50(.80)	-.61	.54	**4.30(1.04)**	**4.67(.49)**	**-2.13***	**.04***
Positive Influencing Factors for CPS-GT Decision-Making								
Number of family members with cancer	4.12(.78)	3.77(1.34)	1.14*	.26	4.09(.90)	3.40(1.40)	1.84*	.09*
Early age of cancer diagnosis in family members	4.33(.78)	4.05(1.17)	1.01*	.29*	4.36(.81)	3.67(1.23)	2.10*	.05*
Presence of known cancer predisposition syndrome in family member	4.29(.94)	3.82(1.30)	1.86	.07	**4.32(.92)**	**3.40(1.35)**	**2.53***	**.02***
Your childs age at initial cancer diagnosis	4.25(.70)	4.23(.97)	.27	.91	4.29(.71)	4.07(1.03)	1.03	.31
Your childs doctors recommendation for cancer predisposition genetic testing	3.96(.90)	4.32(.65)	-1.75	.08	4.00(.89)	4.33(.62)	-1.39	.17
Distinctive (special or particular) characteristics of your childs cancer	4.31(.75)	4.32(.89)	-.02	.98	4.32(.75)	4.20(1.01)	.54	.59
Potential impact on further treatment for your child	4.66(.59)	4.50(.60)	1.09	.28	4.64(.58)	4.47(.64)	1.05	.30
Potential impact on future surveillance for your child	4.61(.62)	4.68(.48)	-.50	.62	4.61(.61)	4.73(.46)	-.77	.45
It is important to have as much information as possible about cancer predispositions for your childs future	**4.61(.77)**	**4.86(.35)**	**-2.14***	**.04***	**4.62(.74)**	**4.93(.26)**	**-2.87***	**.01***
I want to know why my child has developed cancer, regardless of practical impact	**4.22(1.00)**	**4.73(.55)**	**-3.04***	**.003***	**4.22(.98)**	**4.87(.35)**	**-4.48***	**<.001***
Negative Influencing Factors for CPS-GT Decision-Making								
Cancer predisposition genetic testing is not helpful	**2.27(1.42)**	**3.73(1.52)**	**-4.11**	**<.001**	**2.36(1.46)**	**4.20(1.21)**	**-5.19***	**<.001***
Cancer predisposition genetic testing may cause psychological distress to me	**2.88(1.28)**	**3.64(1.36)**	**-2.37**	**.02**	**2.84(1.21)**	**4.00(1.13)**	**-3.19**	**.002**
Cancer predisposition genetic testing may cause psychological distress to my child	3.53(1.13)	3.771.27)	-.85	.40	3.47(1.21)	4.13(.99)	-2.00	.05
Cancer predisposition genetic testing may cause psychological distress to the patient's family	3.39(1.17)	3.73(1.28)	-1.15	.25	**3.34(1.23)**	**4.07(1.03)**	**-2.15**	**.03**
Patient confidentiality/privacy will be at risk	3.04(1.25)	3.32(1.25)	-.89	.37	3.11(1.21)	3.40(1.06)	-.82	.42
Cancer predisposition genetic testing is too costly	3.00(1.37)	3.59(1.05)	-1.85	.07	**3.00(1.06)**	**3.80(.94)**	**-2.16**	**.03**
Patients with positive test results may face discrimination at work or when seeking insurance	3.43(1.25)	3.27(1.45)	.49	.62	3.35(1.29)	3.47(1.46)	-.33	.75
A positive result in cancer predisposition genetic testing may negatively affect relationships with patient's family and relatives	2.74(1.27)	3.14(1.39)	-1.24	.22	2.73(1.29)	3.27(1.39)	-1.46	.15

* Adjusted for heterogeneity of variance using Welch's t-test

Supplementary Material S3. Means and Statistical Findings for Income and Insurance (bolded text indicates significant findings with $p < .05$)

Question	Income				Insurance			
	<$10,000 - <$75,000 (N = 43) Mean	$75,000+ (N = 43) Mean	T-value	P-value	Private insurance (N = 43) Mean	Medicaid (N = 47) Mean	T-value	P-value
Knowledge, Interest, and Parent Versus Child Roles								
I am aware that genetic testing can be used to diagnose cancer predispositions	4.36(.98)	4.61(.77)	-1.30	.20	4.63(.80)	4.30(.92)	1.78	.08
I understand what cancer predisposition genetic testing is	4.28(.91)	4.32(.88)	-.20	.85	4.34(.94)	4.21(.88)	.66	.51
I am in favor of cancer predisposition genetic testing for children and adolescents less than 18 years of age	4.47(.86)	4.49(.68)	-.14	.89	4.54(.64)	4.43(.85)	.68	.50
I am in favor of cancer predisposition genetic testing for children, even when there is no prevention, treatment or cure for the cancer predisposition	4.28(.96)	4.10(.97)	.86	.20	4.20(.95)	4.15(.96)	.23	.82
I am interested in taking a cancer predisposition genetic test for myself	4.49(.77)	4.32(.82)	.99	.16	4.41(.81)	4.32(.84)	.54	.59
I am interested in having my child take a cancer predisposition genetic test	4.51(.77)	4.51(.77)	.15	.45	4.59(.59)	4.40(.80)	1.20	.24
The benefits of cancer predisposition genetic testing outweigh the risks	4.19(1.01)	4.27(.78)	-.42	.34	4.34(.76)	4.00(1.06)	1.71	.09
Parents should decide if children and adolescents less than 18 years of age should be tested for cancer predispositions	4.35(.92)	4.40(.63)	-.33	.37	4.33(.92)	4.38(.66)	-.27	.79
Teenagers above the age of 13 (thirteen) should be involved in making decisions for cancer predisposition genetic testing and communication of results	3.88(1.18)	3.88(1.18)	.68	.25	3.74(1.01)	3.94(1.24)	-.82	.42
Children aged 7-13 (seven to thirteen) should be involved in making the decision for cancer predisposition genetic testing and communication of results	3.30(1.34)	2.76(1.10)	2.03	.05	2.81(1.07)	3.28(.97)	-1.81*	.07*
If children and adolescents less than 18 years of age are tested and they turn out to carry a cancer predisposition, they should be told about the test results immediately	**4.14(1.01)**	**3.55(1.09)**	**2.60**	**.01**	**3.48(.97)**	**4.17(1.11)**	**-3.13**	**.002**
If children and adolescents less than 18 years of age are tested and they turn out not to carry a cancer predisposition, they should be told about the test results immediately	4.40(.98)	4.07(.89)	1.59	.12	**4.05(.91)**	**4.47(.86)**	**-2.25**	**.03**
Pediatricians of tested children and adolescents less than 18 years of age who turn out to carry a cancer predisposition should be told about the results	4.51(.86)	4.26(.99)	1.25	.22	4.29(.89)	4.45(1.02)	-.79	.43
Positive Influencing Factors for CPS-GT Decision-Making								
Number of family members with cancer	3.93(1.16)	4.05(.91)	-.52	.61	4.05(.87)	3.87(1.16)	.82	.42
Early age of cancer diagnosis in family members	4.28(.96)	4.31(.87)	-.15	.88	4.37(.79)	4.11(1.04)	1.34	.18
Presence of known cancer predisposition syndrome in family member	4.26(1.07)	4.14(1.04)	.51	.61	4.19(1.03)	4.11(1.09)	.36	.72
Your child's age at initial cancer diagnosis	4.33(.84)	4.27(.63)	.35	.73	4.26(.67)	4.22(.87)	.27	.79
Your child's doctor's recommendation for cancer predisposition genetic testing	4.16(.84)	3.95(.83)	1.16	.25	3.86(.83)	4.20(.86)	-1.87	.07
Distinctive (special or particular) characteristics of your childs cancer	4.35(.81)	4.33(.75)	.14	.89	4.30(.71)	4.28(.88)	.15	.88
Potential impact on further treatment for your child	4.58(.59)	4.60(.62)	-.18	.86	4.58(.63)	4.64(.57)	-.45	.65
Potential impact on future surveillance for your child	4.69(.52)	4.52(.67)	1.27*	.21*	**4.49(.67)**	**4.78(.42)**	**-2.42***	**.02***
It is important to have as much information as possible about cancer predispositions for your childs future	4.81(.39)	4.69(.60)	1.11*	.27*	4.67(.61)	4.66(.79)	.10	.92
I want to know why my child has developed cancer, regardless of practical impact	4.47(.83)	4.31(.90)	.83	.41	4.28(.85)	4.38(1.03)	-.52	.61
Negative Influencing Factors for CPS-GT Decision-Making								
Cancer predisposition genetic testing is not helpful	**3.29(1.57)**	**1.93(1.23)**	**4.39**	**<.001**	**1.86(1.20)**	**3.45(1.54)**	**-5.09***	**<.001***
Cancer predisposition genetic testing may cause psychological distress to me	3.19(1.45)	2.70(1.10)	1.76*	.08*	**2.70(1.17)**	**3.34(1.46)**	**-2.29**	**.02**
Cancer predisposition genetic testing may cause psychological distress to my child	3.56(1.26)	3.51(1.28)	.18	.86	3.47(1.05)	3.68(1.34)	-.85	.40
Cancer predisposition genetic testing may cause psychological distress to the patients family	3.51(1.28)	3.36(1.14)	.59	.56	3.33(1.12)	3.53(1.32)	-.76	.45
Patient confidentiality/privacy will be at risk	3.17(1.31)	3.05(1.25)	.43	.67	2.95(1.17)	3.28(1.31)	-1.22	.22
Cancer predisposition genetic testing is too costly	3.37(1.35)	2.86(1.28)	1.80	.08	2.86(1.25)	3.26(1.38)	-1.42	.16
Patients with positive test results may face discrimination at work or when seeking insurance	3.19(1.47)	3.44(1.18)	-.89	.38	3.30(1.23)	3.40(1.41)	-.37	.72
A positive result in cancer predisposition genetic testing may negatively affect relationships with patient's family and relatives	2.81(1.39)	2.79(1.19)	.08	.93	2.70(1.15)	2.85(1.43)	-.56	.58

*Adjusted for heterogeneity of variance using Welch's t-test

Supplementary Material S4. Means and Statistical Findings for Employment and Education (bolded text indicates significant findings with $p < .05$

Question	Employment				Education			
	Unemployed (N = 43) Mean	Employed (N = 51) Mean	T-value	P-value	Below 4-year university education (N = 58) Mean	4-year university and above (N = 36) Mean	T-value	P-value
Knowledge, Interest, and Parent Versus Child Roles								
I am aware that genetic testing can be used to diagnose cancer predispositions	4.49(.77)	4.48(.95)	.05	.96	**4.35(1.00)**	**4.69(.53)**	**-2.17***	**.03***
I understand what cancer predisposition genetic testing is	4.28(.91)	4.31(.90)	-.14	.89	4.25(.92)	4.36(.87)	-.58	.57
I am in favor of cancer predisposition genetic testing for children and adolescents less than 18 years of age	4.49(.80)	4.49(.71)	-.01	.99	4.46(.83)	4.53(.61)	-.40	.69
I am in favor of cancer predisposition genetic testing for children, even when there is no prevention, treatment or cure for the cancer predisposition	4.16(1.02)	4.22(.87)	-.31	.76	4.23(.95)	4.14(.93)	.46	.65
I am interested in taking a cancer predisposition genetic test for myself	4.40(.85)	4.37(.78)	.17	.87	**4.52(.74)**	**4.17(.88)**	**2.07**	**.04**
I am interested in having my child take a cancer predisposition genetic test	4.47(.80)	4.53(.62)	-.44	.66	4.52(.74)	4.47(.65)	.30	.76
The benefits of cancer predisposition genetic testing outweigh the risks	4.05(1.07)	4.31(.80)	-1.33	.19	4.18(1.03)	4.19(.79)	-.08	.94
Parents should decide if children and adolescents less than 18 years of age should be tested for cancer predispositions	4.23(.95)	4.54(.58)	-1.85*	.07*	4.34(.84)	4.49(.69)	-.89	.38
Teenagers above the age of 13 (thirteen) should be involved in making decisions for cancer predisposition genetic testing and communication of results	4.00(1.07)	3.70(1.18)	1.28	.21	3.75(1.24)	3.97(.96)	-.93	.36
Children aged 7-13 (seven to thirteen) should be involved in making the decision for cancer predisposition genetic testing and communication of results	**3.37(1.20)**	**2.70(1.22)**	**2.68**	**.01**	2.98(1.29)	3.05(1.20)	-.27	.79
If children and adolescents less than 18 years of age are tested and they turn out to carry a cancer predisposition, they should be told about the test results immediately	**4.12(1.07)**	**3.64(1.06)**	**2.14**	**.04**	**4.09(1.03)**	**3.51(1.10)**	**2.58**	**.01**
If children and adolescents less than 18 years of age are tested and they turn out not to carry a cancer predisposition, they should be told about the test results immediately	**4.51(.77)**	**4.06(1.00)**	**2.42**	**.02**	4.41(.89)	4.05(.94)	1.85	.07
Pediatricians of tested children and adolescents less than 18 years of age who turn out to carry a cancer predisposition should be told about the results	4.47(.91)	4.28(1.03)	.91	.36	4.43(.99)	4.27(.96)	.76	.45
Positive Influencing Factors for CPS-GT Decision-Making								
Number of family members with cancer	4.00(1.06)	3.96(.99)	.19	.85	3.93(1.05)	4.05(.97)	-.59	.56
Early age of cancer diagnosis in family members	4.16(.97)	4.32(.87)	-.82	.41	4.25(.91)	4.24(.94)	.09	.93
Presence of known cancer predisposition syndrome in family member	4.05(1.11)	4.27(.98)	-1.06	.29	4.23(.97)	4.08(1.15)	.70	.49
Your child's age at initial cancer diagnosis	4.14(.83)	4.35(.69)	-1.30	.20	4.19(.83)	4.34(.67)	-.97	.34
Your child's doctor's recommendation for cancer predisposition genetic testing	4.02(.89)	4.08(.83)	-.32	.75	4.15(.80)	3.92(.91)	1.25	.21
Distinctive (special or particular) characteristics of your childs cancer	4.26(.88)	4.35(.72)	-.59	.56	4.25(.82)	4.39(.76)	-.87	.39
Potential impact on further treatment for your child	4.67(.52)	4.57(.64)	.87	.19	4.54(.63)	4.74(.50)	-1.71*	.09*
Potential impact on future surveillance for your child	4.72(.50)	4.55(.65)	1.41*	.16*	4.57(.66)	4.71(.46)	-1.17*	.25*
It is important to have as much information as possible about cancer predispositions for your childs future	4.65(.81)	4.70(.58)	-.34	.37	4.77(.47)	4.54(.93)	1.38*	.18*
I want to know why my child has developed cancer, regardless of practical impact	4.28(1.03)	4.38(.86)	-.52	.61	4.45(.81)	4.16(1.09)	1.36*	.18*
Negative Influencing Factors for CPS-GT Decision-Making								
Cancer predisposition genetic testing is not helpful	2.88(1.61)	2.44(1.53)	1.36	.18	**3.07(1.56)**	**2.03(1.38)**	**3.28**	**<.001**
Cancer predisposition genetic testing may cause psychological distress to me	3.26(1.35)	2.84(1.32)	1.50	.14	3.00(1.39)	3.08(1.28)	-.28	.78
Cancer predisposition genetic testing may cause psychological distress to my child	3.70(1.10)	3.47(1.27)	.92	.36	3.55(1.24)	3.61(1.15)	-.21	.84
Cancer predisposition genetic testing may cause psychological distress to the patients family	3.58(1.12)	3.36(1.31)	.87	.39	3.45(1.25)	3.47(1.20)	-.07	.94
Patient confidentiality/privacy will be at risk	3.12(1.26)	3.18(1.29)	-.25	.80	3.18(1.22)	3.11(1.35)	.27	.79
Cancer predisposition genetic testing is too costly	3.35(1.27)	2.94(1.36)	1.49	.14	**3.36(1.30)**	**2.76(1.30)**	**2.24**	**.03**
Patients with positive test results may face discrimination at work or when seeking insurance	3.37(1.29)	3.37(1.29)	-.07	.94	3.36(1.34)	3.42(1.29)	-.23	.82
A positive result in cancer predisposition genetic testing may negatively affect relationships with patient's family and relatives	2.81(1.28)	2.84(1.35)	-.11	.92	2.88(1.25)	2.76(1.40)	.41	.69

*Adjusted for heterogeneity of variance using Welch's t-test

Supplementary Material S5. Means and Statistical Findings for Parent versus Patient Dyads

Question	Parent Vs. Patient			
	Parent (N = 19) Mean	Patient (N = 19) Mean	T-Value	P-Value
Knowledge, Interest, and Parent Versus Child Roles				
I am aware that genetic testing can be used to diagnose cancer predispositions	4.47(1.02)	4.21(.54)	1.00	.33
I understand what cancer predisposition genetic testing is	**4.47(.61)**	**3.74(.87)**	**3.02**	**.01**
I am in favor of cancer predisposition genetic testing for children and adolescents less than 18 years of age	4.42(.69)	4.00(.81)	1.91	.06
I am in favor of cancer predisposition genetic testing for children, even when there is no prevention, treatment or cure for the cancer predisposition	**4.26(.81)**	**3.58(.96)**	**2.38**	**.02**
I am interested in taking a cancer predisposition genetic test for myself	4.37(.76)	3.95(.62)	1.87	.07
The benefits of cancer predisposition genetic testing outweigh the risks	**4.26(.73)**	**3.58(.96)**	**2.38**	**.02**
Parents should decide if children and adolescents less than 18 years of age should be tested for cancer predispositions	**4.37(.68)**	**3.37(1.12)**	**11.09***	**.002**
Teenagers above the age of 13 (thirteen) should be involved in making decisions for cancer predisposition genetic testing and communication of results	4.21(.92)	4.05(.71)	.60	.56
Children aged 7-13 (seven to thirteen) should be involved in making the decision for cancer predisposition genetic testing and communication of results	2.95(1.22)	3.42(.96)	-1.33	.19
If children and adolescents less than 18 years of age are tested and they turn out to carry a cancer predisposition, they should be told about the test results immediately	4.21(.71)	4.26(.73)	-.22	.82
If children and adolescents less than 18 years of age are tested and they turn out not to carry a cancer predisposition, they should be told about the test results immediately	4.26(.99)	4.44(.71)	-.64	.53
Pediatricians of tested children and adolescents less than 18 years of age who turn out to carry a cancer predisposition should be told about the results	4.26(1.10)	4.47(.61)	-.73	.47
Positive Influencing Factors for CPS-GT Decision-Making				
Number of family members with cancer	4.16(1.12)	4.00(.94)	.47	.64
Early age of cancer diagnosis in family members	4.05(1.22)	3.84(.90)	.61	.55
Presence of known cancer predisposition syndrome in family member	4.26(1.24)	4.00(1.00)	.72	.48
Child's age at initial cancer diagnosis	4.26(.65)	3.79(.92)	1.83	.08
Child's doctors recommendation for cancer predisposition genetic testing	3.89(.74)	3.58(.90)	1.18	.25
Distinctive (special or particular) characteristics of child's cancer	4.47(.70)	3.95(.91)	2.00	.05
Potential impact on further treatment for your child	**4.63(.60)**	**4.05(1.03)**	**2.13**	**.04**
Potential impact on future surveillance for your child	4.56(.71)	4.11(.66)	2.01	.05
Negative Influencing Factors for CPS-GT Decision-Making				
Cancer predisposition genetic testing is not helpful	3.00(1.80)	2.37(1.17)	1.23*	.23*
Cancer predisposition genetic testing may cause psychological distress to me	3.50(1.34)	2.95(1.08)	1.39	.18
Cancer predisposition genetic testing may cause psychological distress to the patients family	3.84(1.21)	3.21(.92)	1.81	.08
Patient confidentiality/privacy will be at risk	3.41(1.23)	3.21(1.08)	.52	.61
Cancer predisposition genetic testing is too costly	3.58(1.54)	3.16(.83)	1.05*	.30*
Patients with positive test results may face discrimination at work or when seeking insurance	3.47(1.17)	3.28(1.07)	.53	.60
A positive result in cancer predisposition genetic testing may negatively affect relationships with patient's family and relatives	3.42(1.31)	2.84(1.12)	1.47	.15

* Adjusted for heterogeneity of variance using Welch’s t-test
